# Effect of Baicalein on the Pharmacokinetics of Cilostazol and Its Two Metabolites in Rat Plasma Using UPLC-MS/MS Method

**DOI:** 10.3389/fphar.2022.888054

**Published:** 2022-04-27

**Authors:** Qinghua Weng, Chaojie Chen, Jianhua Xiong, Ya-Nan Liu, Xinxin Pan, Ju Cui, Jian-Ping Cai, Ren-Ai Xu

**Affiliations:** ^1^ The Third Affiliated Hospital of Shanghai University (Wenzhou People’s Hospital), Wenzhou, China; ^2^ The First Affiliated Hospital of Wenzhou Medical University, Wenzhou, China; ^3^ School of Pharmacy, Wenzhou Medical University, Wenzhou, China; ^4^ The Key Laboratory of Geriatrics, Beijing Institute of Geriatrics, Institute of Geriatric Medicine, Chinese Academy of Medical Sciences, Beijing Hospital/National Center of Gerontology of National Health Commission, Beijing, China

**Keywords:** cilostazol, 3, 4-dehydro cilostazol, 4′-trans-hydroxy cilostazol, baicalein, pharmacokinetics, UPLC-MS/MS

## Abstract

This study aimed to explore the effect of baicalein on the pharmacokinetics of cilostazol (CLZ) and its two metabolites 3,4-dehydro cilostazol (3,4-CLZ) and 4′-trans-hydroxy cilostazol (4′-CLZ) in rats using a newly established ultra performance liquid chromatography tandem mass spectrometry (UPLC-MS/MS) method. Ticagrelor was used as an internal standard (IS), then cilostazol and its two metabolites were separated by means of a UPLC BEH C18 column (2.1 mm × 50 mm, 1.7 μm) using gradient elution method with 0.4 ml/min of flow rate. Acetonitrile as organic phase and water with 0.1% formic acid as aqueous phase constructed the mobile phase. Selective reaction monitoring (SRM) mode and positive ion mode were preferentially chosen to detect the analytes. Twelve SD rats were divided into two groups (*n* = 6) when CLZ was administered orally (10 mg/kg) with or without oral baicalein (80 mg/kg). The selectivity, linearity, recovery, accuracy, precision, matrix effect and stability of UPLC-MS/MS assay were satisfied with the standards of United States Food and Drug Administration guidelines. In control group, AUC_0-∞_ and C_max_ of CLZ were 2,169.5 ± 363.1 ng/ml^*^h and 258.9 ± 82.6 ng/ml, respectively. The corresponding results were 3,767.6 ± 1,049.8 ng/ml^*^h and 308.6 ± 87.9 ng/ml for 3, 4-CLZ, 728.8 ± 189.9 ng/ml^*^h and 100.3 ± 51.3 ng/ml for 4′-CLZ, respectively. After combination with baicalein, AUC_0-∞_ and C_max_ of CLZ were 1.48, 1.38 times higher than the controls. Additionally, AUC_0-∞_ and C_max_ were separately decreased by 36.12 and 19.54% for 3,4-CLZ, 13.11 and 44.37% for 4′-CLZ. Baicalein obviously alters the pharmacokinetic parameters of CLZ, 3,4-CLZ and 4′-CLZ in rats. These results suggested that there was a potential drug-drug interaction between baicalein and CLZ. Therefore, it must raise the awareness when concomitant use of CLZ with baicalein, the dosage regimen of CLZ should be taken into consideration, if this result is confirmed in clinical studies.

## Introduction

*Scutellaria baicalensis*, is widely used as a medicinal material in China for thousands of years. The roots of *Scutellaria baicalensis* called Huangqin were reported to have various pharmacological effects reviewed in a previous study (Zhao et al., 2016), including anticancer functions, hepatoprotection, antibacterial and antiviral activities. Baicalein is one of the main active ingredients of Huangqin. Mounting evidences showed wide pharmacological functions such as alleviation of oxidative stress and inflammation (Yan et al., 2020), suppression of cancer (He et al., 2021), protection of liver injury (Dai et al., 2021). According to the literature of Bai, C. et al., the annual yield of *Scutellaria baicalensis* is over 10,000 tons, and the content of baicalein was approximately 7 mg/g in the dry roots of 2-year-old *Scutellaria baicalensis* (Bai et al., 2020)*.* It was also used as a food condiment in our daily life. In addition, baicalein enhanced the oral bioavailability of tamoxifen, which may be mainly attributable to inhibition of the CYP3A4-mediated metabolism of tamoxifen in rats (Li et al., 2011). Based on various pharmacological effects and wide use of baicalein in clinical applications and potential diet intake, drug-drug interaction with other clinical proved drugs must be investigated.

Cilostazol (CLZ), an anti-platelet drug, is commonly applied as a treatment intervention method in cardiovascular disease, ischemic stroke, diabetes, etc. (Zheng et al., 2019). CLZ was mainly metabolized by hepatic cytochrome P450 such as CYP3A4 and CYP2C19 (Hiratsuka et al., 2007), and 3,4-dehydro cilostazol (3,4-CLZ) and 4′-trans-hydroxy cilostazol (4′-CLZ) were two of the main active metabolites. From the past researches, baicalein might be the substrates of CYP3A4 in the vitro metabolism of SD rat liver microsomes (Zhou et al., 2011), and exhibited an inhibitory effect of CYP3A4 *in vitro* (Meng et al., 2021)*.* Considering the beneficial effects of baicalein on human health, the potential intake of baicalein for patients who take CLZ in daily life may occur. Thus, it is necessary to evaluate drug-drug interaction between baicalein and CLZ.

In the past decades, there have been reported several methods such as high performance liquid chromatography (HPLC), liquid chromatography tandem mass spectrometry (LC-MS/MS) and ultra performance liquid chromatography tandem mass spectrometry (UPLC-MS/MS) for quantitative analysis of CLZ and its metabolites in different species and various biological samples. Previous studies have reported HPLC method of CLZ and its metabolites in various biological samples, including human plasma, human urine, human liver microsomal incubation mixtures (Tata et al., 1998; Fu et al., 1999; Tata et al., 2001). LC-MS/MS method is gradually applied for the detection of CLZ and its metabolites along with mass spectrometry development. Two studies reported UPLC-MS/MS method to simultaneously detect CLZ and 3,4-CLZ in human plasma (Bhatt et al., 2015; Shen et al., 2021). However, only one metabolite detection in human plasma resulted in the application of pharmacokinetic research under restrictions. Until to now, there was no UPLC-MS/MS assay to simultaneously quantify CLZ, 3,4-CLZ and 4′-CLZ in rat plasma and application for pharmacokinetics.

In this experiment, we attempted to develop a rapid, stable and sensitive UPLC-MS/MS method to simultaneously measure the concentrations of CLZ, 3,4-CLZ and 4′-CLZ in rat plasma. Moreover, we used this novel quantitative method for application of the investigation of the pharmacokinetic effect of baicalein on CLZ, 3,4-CLZ and 4′-CLZ in rats.

## Materials and Methods

### Chemicals and Reagents

All chemicals and reagents were showed at Table 1.

**TABLE 1:** List of chemicals and reagents.

Name	Source
3,4-dehydro cilostazol^a^ and 4′-trans-hydroxy cilostazol^a^	Toronto Research Chemicals (Canada)
Cilostazol^a^ and ticagrelor^a^	Shanghai Chuangsai Technology Co., Ltd. (China)
Methanol^b^/Acetonitrile^b^	Merck Company (Germany)
Formic acid^c^	Shanghai Aladdin Biochemical Technology Co., Ltd. (China)
Ultrapure water	Milli-Q Water Purification System (United States of America)

a The purity of chemicals is >98%.

b All reagents are HPLC, grade.

c All reagents are analytical reagent (AR) grade.

### Calibrations Standards, and Quality Control (QC)

Usually, stock solutions were used as mother samples, which could be easily dissolved into a series of the lower concentrations of targets. In this study, we accurately prepared four kinds of stock solutions such as CLZ, 3,4-CLZ, 4′-CLZ and ticagrelor (internal standard, IS) into the final concentrations of 1.00 mg/ml by methanol. Subsequently, work solutions were prepared through the corresponding four stock solutions. Working solutions of CLZ, 3,4-CLZ, 4′-CLZ were dissolved into methanol to prepare the mixture standard working solutions contained three targets with the same final concentrations ranged from 1 to 500 ng/ml. The final concentration of IS working solution diluted with methanol was 200 ng/ml. Finally, the working solutions were used to construct calibrations curve with eight different concentrations of 1, 2, 5, 20, 50, 100, 200, and 500 ng/ml. In the similar method described above, mixture quality control samples (QCs) consisted of CLZ, 3,4-CLZ, 4′-CLZ with four different final concentrations of 1 (lower limit of quantification, LLOQ), 2 (low concentration of quality control sample, LQC), 80 (medium concentration of quality control sample, MQC) and 400 (high concentration of quality control sample, HQC) ng/mL were prepared. All the solutions contained stocking solution and QCs were stored at a refrigerator kept 4°C.

### Animal Experiments

SD rats, weighted from 180 to 220 g, were provided from the Laboratory Animal Center of Wenzhou Medical University (Zhejiang, China). Subsequently, all rats were stayed and kept without special treatments for seven continuous days in the proper environment, such as temperature 25–28°C, humidity 50–60% and 12 h light/12 h dark, etc. During this period, unlimited drinking water and barley-based diets were supplied.

The Institutional Ethics Committee of Wenzhou Medical University (Zhejiang, China) approved this animal experiments, which strictly adhered to the National Institute of Health (NIH) guidelines. The detailed experimental process was showed as follows: firstly, a total of 12 SD rats were equally divided into single group (CLZ) and combination group (baicalein plus CLZ). Water was allowed before the first gavage, but a necessary restriction of food for 12 h was needed. Combination group of rats were orally given baicalein with the concentration of 80 mg/kg. Control ones (single group) were orally administrated with the equal volume of distilled water. Subsequently, 10 mg/kg CLZ was applied for all rats after half an hour. Caudal veins for blood collection were used at different time points of 0, 0.333, 0.667, 1, 1.5, 2, 3, 4, 6, 8, 12, 24, 48 h after the last oral administration. Heparin-containing tubes were used for temporarily storing blood samples, which were further centrifuged at 4,000 × *g* for 8 min to obtain the supernatants as plasma samples.

### Plasma Preparation

A simple process of protein precipitation with acetonitrile was finally supplied for direct plasma preparation. A complete plasma preparation was consisted of three parts, including addition of IS, access to precipitant and centrifuge for the acquisition of the supernatants. Firstly, added 20 μl ticagrelor as IS into 100 μl of plasma samples, then 300 μl acetonitrile as precipitant was added into the mixture above. Subsequently, we centrifuged after adequately vortexing. Finally, 100 μl of the supernatants was obtained for the further UPLC-MS/MS quantitative analysis.

### UPLC-MS/MS Analytical Conditions

CLZ and its metabolites were separated and detected by a Waters ACQUITY UPLC I-Class system and a Xevo TQ-S triple quadrupole tandem mass spectrometer equipped with an electro-spray ionization (ESI) source (Milford, MA, United States). The temperature of column and autosampler were 40 and 10°C, respectively. 2.0 µl of injection volume was pushed into UPLC-MS/MS system and 0.40 ml/min of total flow rate was employed for the further analysis. Gradient elution consisted of solvent A (acetonitrile) and solvent B (0.1% formic acid in water) was performed on a Acquity UPLC BEH C18 column (2.1 mm × 50 mm, 1.7 μm). The detailed elution condition was as follows: 0–0.5 min, 90% B; 0.5–1 min, 90%–10% B; 1–1.5 min, 10% B; 1.5–1.6 min, 10%–90% B; 1.6–2.0 min, 90% B.

Selective reaction monitoring (SRM) mode and positive ion mode was used to determine the levels of three targets and IS. In this study, ion transitions of CLZ, 3,4-CLZ and 4′-CLZ for quantitative detection were *m/z* 370.30 → 125.02, *m/z* 368.20 → 286.12, and *m/z* 386.20 → 288.16, respectively. The detailed mass spectrometer conditions were showed at Table 2. To collect and further process the experimental data, the system-provided Quanlynx programme and Masslynx 4.1 software (Milford, MA, United States) were needed.

**TABLE 2:** Parameters of mass spectrometer.

Parameters		Detailed value
Flow rate	Collision gas	0.15 ml/min
	Cone gas	200 L/h
	Desolvation gas	1000 L/h
Collision energy	Cilostazol	20 eV
	3,4-dehydro cilostazol	15 eV
	4′-trans-hydroxy cilostazol	13 eV
Cone voltage	cilostazol	30 V
	3,4-dehydro cilostazol	20 V
	4′-trans-hydroxy cilostazol	20 V
Desolvation temperature		600°C
Capillary voltage		2.0 kV

### Method Validation

UPLC-MS/MS method has been a homeliness and practical approach applied for the determination of biological specimens. In this research, a rapid, stable and sensitive UPLC-MS/MS method was established and verified for selectivity, sensitivity, linearity, LLOQ, matrix effect, recovery, accuracy, precision and stability. To accurately and legitimately detect three targets, the method validation strictly adhered to the United States Food and Drug Administration (FDA) guidelines (U.S. Department of Health and Human Services Food and Drug Administration (FDA), Center for Drug Evaluation and Research (CDER), Center for Veterinary Medicine (CVM), Bioanalytical Method Validation Guidance for Industry, Center for Drug Evaluation and Research, 2018, https://www.fda.gov/regulatory-information/search-fda-guidance-documents/bioanalytical-method-validation-guidance-industry).

Selectivity was mainly to check the absence of interferences at the nearby retention times of three targets and IS using six replicates samples. Three kinds of analytic specimens such as blank rat plasma, the mixture standard solution of three targets at LLOQ concentrations and real rat plasma after oral administration of CLZ. Sensitivity was scientifically estimated by the value of the lowest quantitative point, namely LLOQ, which was finally investigated at the condition of 10 times of signal-to-noise ratio (S/N). In this study, calibration curves of the analytes were evaluated by eight values identified as the ratio of peak area (analyte/IS) against the nominal concentrations of the analyte using a weighted (1/×) least square regression model.

Precision, accuracy, recovery, and matrix effect were eventually estimated by six replicates specimens using QCs in three different levels (2, 80, 400 ng/ml) of CLZ, 3,4-CLZ and 4′-CLZ. In addition, 1 ng/ml of CLZ, 3,4-CLZ and 4′-CLZ was also used to assess precision and accuracy. Relative standard deviation (RSD%) and relative error (RE%) were separately used to evaluate accuracy and precision. Precisions divided into intra-precision and inter-precision were detected on the same day and three continuous days, respectively. Recovery was recognized as the peak area of CLZ or 3,4-CLZ or 4′-CLZ before and after plasma preparation. Matrix effect of CLZ or 3,4-CLZ or 4′-CLZ was investigated by comparative study between the peak area of the spike-after-extraction samples and the corresponding results of the pure solution.

Stability was estimated by six replicates specimens using QCs in three different levels (2, 80, 400 ng/ml). The stability evaluation of CLZ or 3,4-CLZ or 4′-CLZ was performed under different environmental conditions composed of temperature and perservation time, including room temperature (2 h), −80°C (3 weeks), 10 °C in an autosampler (4 h after preparation) and three freeze/thaw cycles (−80°C to room temperature).

### Statistical Analysis

Drug and Statistics (DAS) 3.0 software, produced by Mathematical Pharmacology Professional Committee of China, was eventually used to analyze and calculate the pharmacokinetic parameters of CLZ and its main metabolites *via* a non-compartmental method. Furthermore, unpaired *t*-test analysis applied for difference detection was performed in SPSS 17.0, where *p* < 0.05 was considered as the obvious criteria.

## Results

### Method Development and Optimization

**FIGURE 1:**
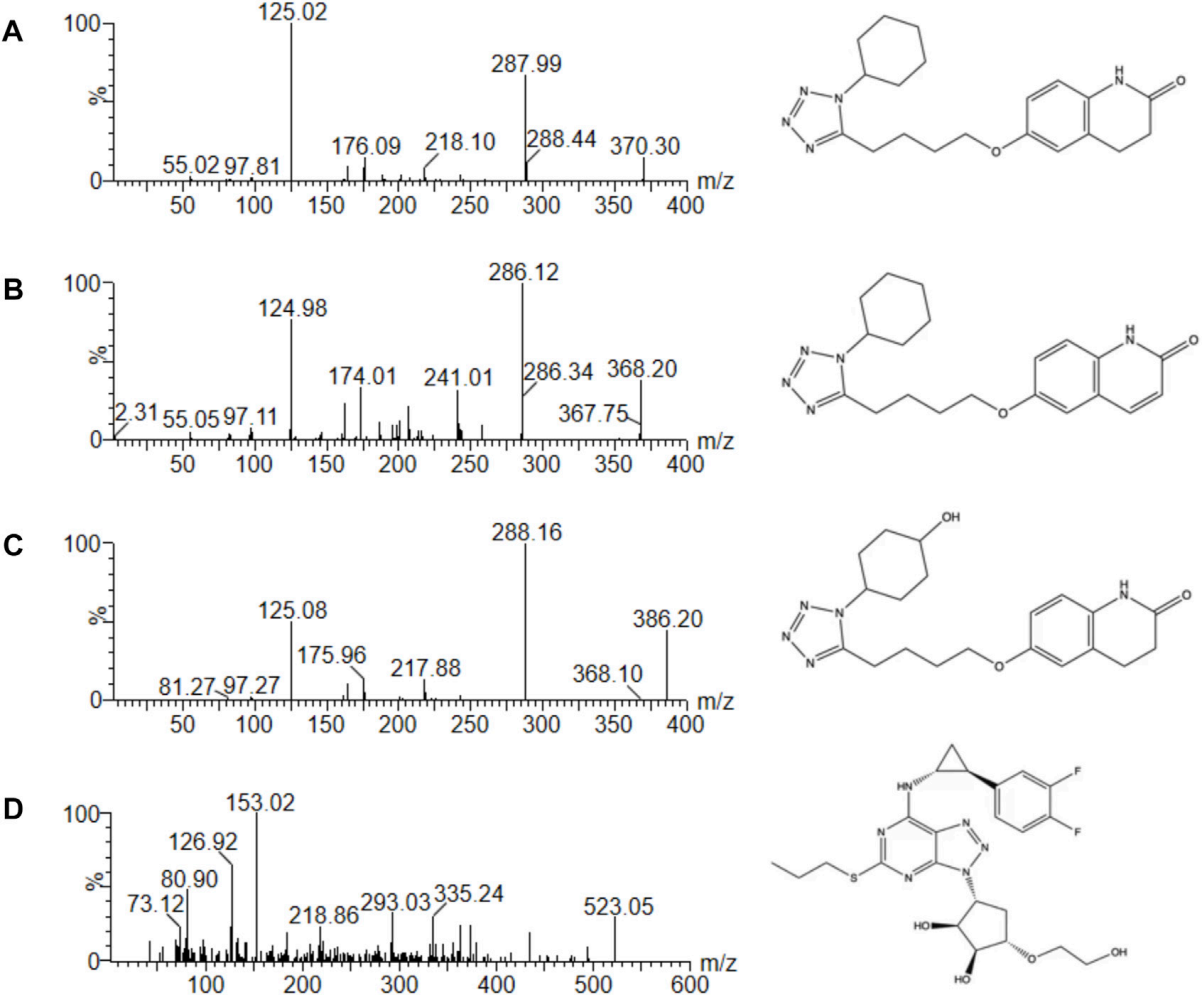
Mass spectra of cilostazol **(A)**, 3,4-dehydro cilostazol **(B)**, 4′-trans-hydroxy cilostazol **(C)** and IS **(D)** in this study.

To get the better analytic results, we optimized the MS parameters and acquired the better settings using CLZ, 3,4-CLZ, 4′-CLZ and IS. Figure 1 was mass spectrum of CLZ, 3,4-CLZ, 4′-CLZ and IS. The parent ion of CLZ, 3,4-CLZ, 4′-CLZ was *m/z* 370.30, 368.20, 386.20, respectively, and the corresponding ion of IS was *m/z* 523.05. The most abundant fragment ion at *m/z* 125.02, 286.12, 288.16, and 153.02 separately belonged to CLZ, 3,4-CLZ, 4′-CLZ and IS, which were chosen as the product ions for the bioanalytical method.

The bioanalytical assay was developed in several parts such as LLOQ, time consumption and injection volume compared with other quantitative methods in rat plasma. According to the previous study, the LLOQ of CLZ and 3,4-CLZ were 20 ng/ml (Varanasi et al., 2008). In this research, the LLOQ of CLZ, 3,4-CLZ and 4′-CLZ were low as 1.0 ng/ml. Time consumption of the chemical separation and detection was only 2.0 min. However, it needed 1.75 times consumption described by Varanasi, K. K. et al. (Varanasi et al., 2008) with the time of 3.5 min. 2 μl was adequate volume in this work, while the corresponding was 20 μl reported by Zhou, H. et al. (Zhou et al., 2021). Importantly, the additional analysis of bioactive metabolite 4′-CLZ in rat plasma was never reported before.

### Method Validation

#### Selectivity

**FIGURE 2:**
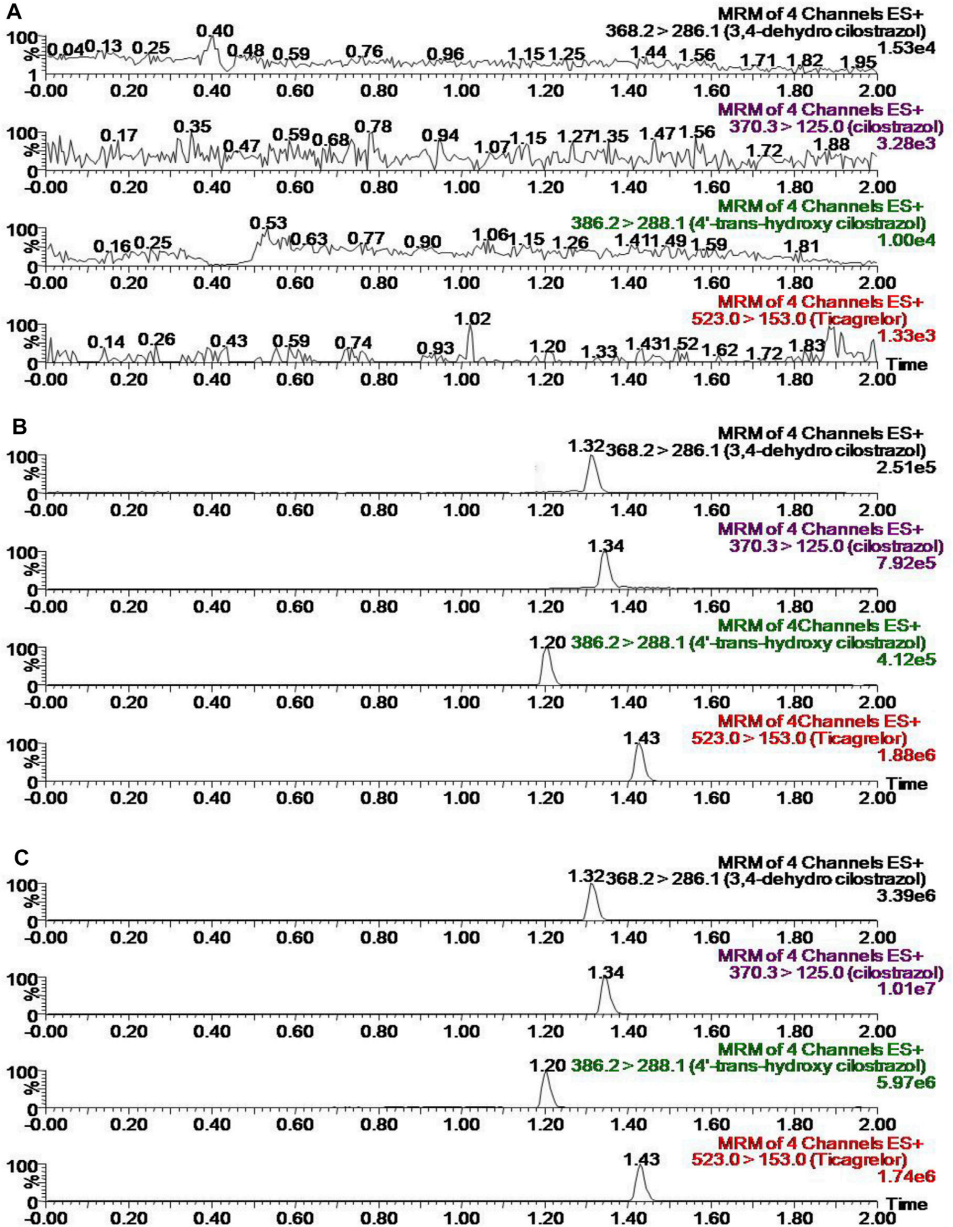
Representative chromatograms of cilostazol, its metabolites and IS in rat plasma **(A)** blank plasma **(B)** blank plasma spiked with standard solutions **(C)** sample from a rat at 1.0 h after gavage with 10 mg/kg cilostazol and a subsequent standard plasma preparation.

Three kinds of biological samples such as blank plasma, blank plasma with standard solutions at LLOQ concentrations and plasma specimens with standard preparations after gavage administration of CLZ were applied for estimating selectivity. The final results were listed at Figure 2. In this work, we found no potential endogenous and external substances at the nearby retention times of CLZ, 3,4-CLZ, 4′-CLZ and IS. The results from above indicated that interference factor-related quantitative analysis of CLZ, 3,4-CLZ and 4′-CLZ didn’t appear in this methodology.

#### Calibration Curve and LLOQ

In this research, we used eight different concentrations to construct calibration curves of CLZ and its two metabolites. Calibration curve of CLZ was Y = 3.27213 × X + 5.07792, Y = 1.80371 × X + 2.14749 for 3,4-CLZ and Y = 1.76848 × X + 1.13212 for 4′-CLZ. Correlation coefficient of three targets reached over 0.99, which represented an excellent linearity. LLOQ of three targets at the concentration of 1 ng/ml was finally validated and accepted in standard ranges.

#### Precision and Accuracy

The parameters of precision and accuracy was described as RSD% and RE%, respectively. Table 3 showed the values of precision and accuracy, both of which were divided into intra- and inter-day. The highest precision of CLZ reached 13.1%, the lowest precision of the corresponding one was 1.8%. Moreover, precision of two metabolites ranged from 2.1 to 14.4%. Meanwhile, the accuracy of ciloctazol ranged from -7.6 to 5.2%. and the corresponding ones of two metabolites were from -5.6 to 14.8%. Together with these results implicated that the precision and accuracy were approbatory scope of FDA guidelines.

**TABLE 3:** Precision and accuracy of cilostazol and its metabolites in rat plasma (*n* = 6).

Analytes	Concentration (ng/ml)	Intra-day		Inter-day	
		RSD%	RE%	RSD%	RE%
Cilostazol	1	11.9	-7.4	13.1	-7.6
	2	10.1	-3.1	11.0	-2.4
	80	4.4	1.2	9.1	-0.4
	400	1.8	4.4	2.2	5.2
3,4-dehydro cilostazol	1	11.3	14.8	14.4	2.8
	2	7.1	14.3	8.9	10.4
	80	6.4	2.8	7.4	2.6
	400	2.1	8.9	2.9	8.9
4′-trans-hydroxy cilostazol	1	6.1	-5.6	7.7	-0.5
	2	5.7	2.1	6.4	8.9
	80	3.1	2.0	3.1	5.0
	400	2.1	5.9	2.5	7.2

#### Recovery and Matrix Effect

Table 4 represented the values of recovery and matrix effect for CLZ and its two metabolites. From the described results, splendid recoveries and no remarkable matrix effects within the bounds of FDA guidelines were found for CLZ and its two metabolites.

**TABLE 4:** Recovery and matrix effect of cilostazol and its metabolites in rat plasma (*n* = 6).

Analyte	Concentration (ng/ml)	Recovery (%)		Matrix effect (%)	
		Mean ± SD	RSD (%)	Mean ± SD	RSD (%)
Cilostazol	2	78.6 ± 11.1	14.1	100.4 ± 13.5	13.5
	80	81.5 ± 5.1	6.2	94.9 ± 8.0	8.4
	400	84.2 ± 1.2	1.4	98.2 ± 3.4	3.5
3,4-dehydro cilostazol	2	80.7 ± 10.5	13.1	100.0 ± 14.3	14.3
	80	81.4 ± 2.8	3.4	93.1 ± 7.9	7.9
	400	86.1 ± 3.4	4.0	90.5 ± 2.6	2.6
4′-trans-hydroxy cilostazol	2	76.9 ± 2.5	3.2	94.6 ± 12.7	13.4
	80	77.3 ± 11.3	14.6	91.6 ± 11.1	12.1
	400	79.0 ± 6.7	8.5	99.9 ± 3.7	3.7

#### Stability

Stability testing results of CLZ and its two metabolites under different preparation and storage conditions in rat plasma were showed at Table 5. RSD% ranged from 1.9 to 14.9%, and RE% was from -13.6 to 5.1%. The described results showed an excellent stability.

**TABLE 5:** Stability results of cilostazol and its metabolites in plasma under different conditions (*n* = 6).

Analyte	Added (ng/ml)	Room temperature, 2 h		Autosampler 10°C, 4 h		Three freeze-Thaw		-80°C, 3 weeks	
		RSD (%)	RE (%)	RSD (%)	RE (%)	RSD (%)	RE (%)	RSD (%)	RE (%)
Cilostazol	2	14.9	−12.3	12.6	−12.8	13.4	−8.3	7.7	−9.7
	80	13.7	−6.9	5.0	−10.8	11.3	−12.2	2.6	−11.4
	400	4.5	−11.1	2.8	−6.8	2.9	−7.0	2.1	−10.2
3,4-dehydro cilostazol	2	8.7	−4.4	6.7	−12.4	8.8	−13.6	5.8	−3.5
	80	6.8	−11.3	2.7	−8.1	5.3	−9.0	5.6	−12.8
	400	2.7	−12.1	2.6	−4.4	2.5	−5.4	2.4	−7.7
4′-trans-hydroxy cilostazol	2	7.5	−8.3	5.2	−8.7	5.3	4.3	2.4	5.1
	80	6.6	−12.9	3.3	−3.1	3.3	−0.1	11.7	−5.9
	400	3.7	−11.2	2.7	−0.6	2.5	−1.0	1.9	−2.2

#### Animal Study

**FIGURE 3:**
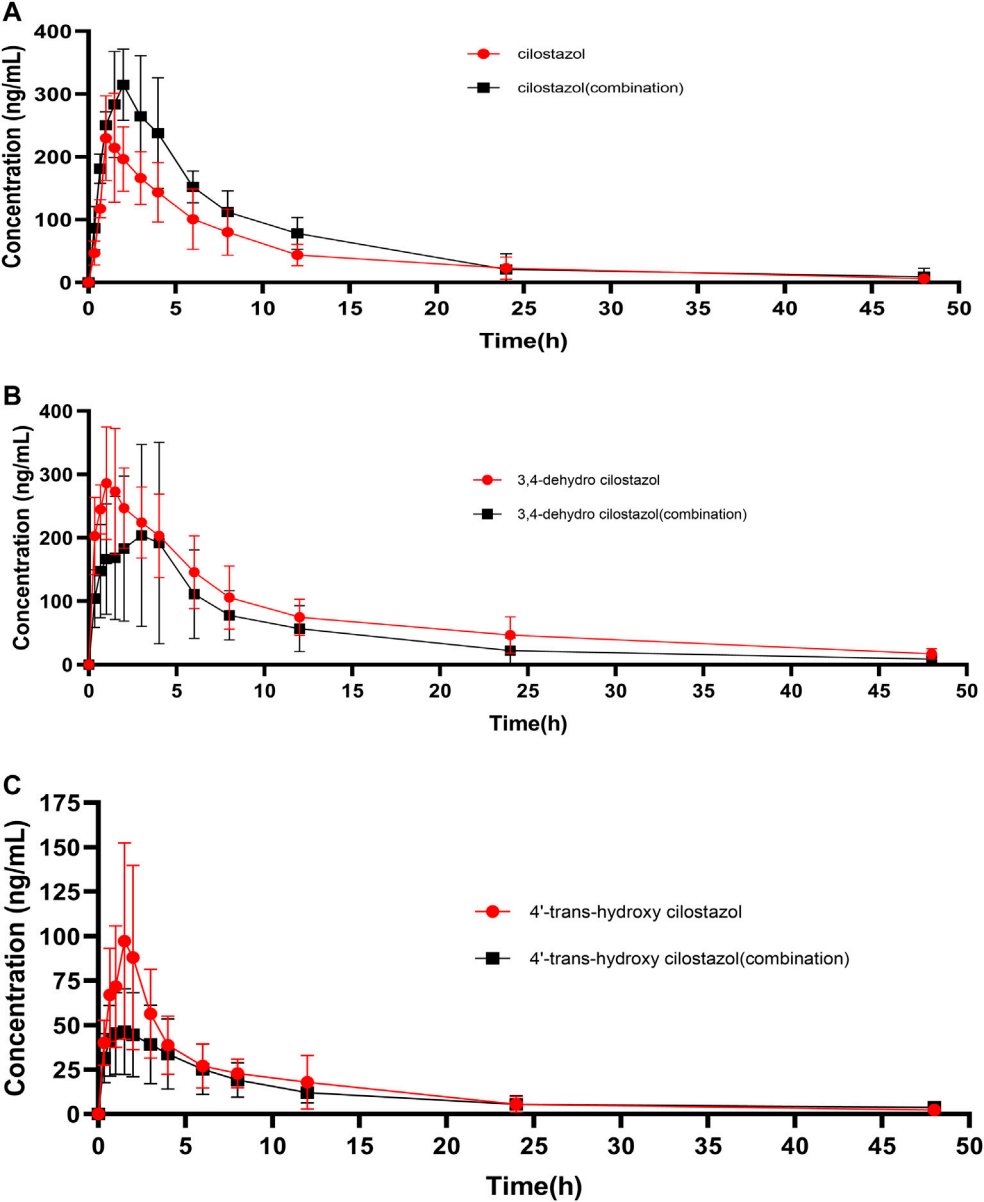
Mean plasma concentration-time curves of cilostazol and its two metabolites in rats after oral administration of 10 mg/kg cilostazol with or without 80 mg/kg baicalein (*n* = 6).

The plasma concentration-time curves of CLZ in rat plasma was showed in Figure 3A after single oral administration of 10 mg/kg CLZ or combination with 80 mg/kg baicalein. Figures 3B,C represented the plasma concentration-time curves of 3,4-CLZ and 4′-CLZ, respectively. DAS program was used to calculate the main parameters of pharmacokinetics such as area under the plasma concentration-time curve (AUC), mean residence time (MRT), elimination half-time (t_1/2_), time to reach the peak plasma concentration (T_max_), and the peak plasma concentration (C_max_) after single CLZ or combinations. All the results were finally filled in Table 5.

#### Pharmacokinetics of CLZ and Its Metabolites Alone

Interestingly, results from Figure 3; Table 6 showed that the plasma levels of CLZ reached its maximum (258.9 ± 82.6 ng/ml) within 1.8 ± 1.2 h, and the corresponding results of 3,4-CLZ and 4′-CLZ were 1.4 ± 0.8 h (308.6 ± 87.9 ng/ml), 3.1 ± 1.4 h (100.3 ± 51.3 ng/ml), respectively. These observations indicated that CLZ was fast absorbed after oral administration and thereafter transformed into two active metabolites. Moreover, t_1/2_ value of CLZ was 10.7 ± 3.4 h, which showed a long t_1/2_. The similar results found in 3,4-CLZ and 4′-CLZ. AUC_(0-∞)_ and CLz/F, Vz/F were 2,169.5 ± 363.1 ng/ml^*^h, 4.8 ± 1.0 L/h/kg and 71.1 ± 18.6 L/kg for CLZ, 3,767.6 ± 1,049.8 ng/ml^*^h, 2.9 ± 1.1 L/h/kg, 60.1 ± 19.2 L/kg for 3,4-CLZ, 728.8 ± 189.9 ng/ml h, 14.4 ± 3.4 L/h/kg and 253.0 ± 118.0 L/kg for 4′-CLZ, respectively.

**TABLE 6:** The main pharmacokinetic parameters of cilostazol and its metabolites with or without baicalein in rat plasma. (*n* = 6, Mean ± SD).

Parameters	Cilostazol		3,4-dehydro cilostazol		4′-trans-hydroxy cilostazol	
	Single	Combination	Single	Combination	Single	Combination
AUC_0→t_ (ng/ml^*^h)	2065.1 ± 316.6	2949.4 ± 628.5^a^	3375.3 ± 857.3	2284.6 ± 1323.0^a^	686.3 ± 171.7	544.9 ± 278.3^a^
AUC_0→∞_ (ng/ml^*^h)	2169.5 ± 363.1	3221.1 ± 1088.0^a^	3767.6 ± 1049.8	2406.6 ± 1366.1^a^	728.8 ± 189.9	633.2 ± 410.0^a^
MRT_0→t_ (h)	10.8 ± 3.3	9.5 ± 4.1	12.6 ± 3.1	10.8 ± 2.8	10.0 ± 3.8	11.7 ± 3.4
MRT_0→∞_ (h)	13.3 ± 4.9	13.5 ± 11.2	18.2 ± 5.5	14.2 ± 5.2	13.3 ± 6.3	18.7 ± 14.3
t_1/2_ (h)	10.7 ± 3.4	10.3 ± 8.8	14.8 ± 3.2	11.0 ± 4.1	12.4 ± 4.9	12.1 ± 4.3
T_max_ (h)	1.8 ± 1.2	2.1 ± 0.5	1.4 ± 0.8	2.4 ± 1.2	3.1 ± 1.4	1.9 ± 0.9^a^
CLz/F (L/h/kg)	4.8 ± 1.0	3.4 ± 1.0^a^	2.9 ± 1.1	6.5 ± 5.4^a^	14.4 ± 3.4	25.5 ± 5.9^a^
Vz/F (L/kg)	71.1 ± 18.6	42.4 ± 12.9^a^	60.1 ± 19.2	102.9 ± 83.9	253.0 ± 118.0	289.7 ± 153.1
C_max_ (ng/ml)	258.9 ± 82.6	357.1 ± 64.7^a^	308.6 ± 87.9	248.3 ± 147.8	100.3 ± 51.3	55.8 ± 26.8^a^

a Represents *p* < 0.05.

#### Effect of Baicalein on the Pharmacokinetics of CLZ and Its Metabolites in Rats

Baicalein obviously increased systemic exposure of CLZ: AUC_0-∞_ (3221.1 ± 1088.0 ng/ml^*^h) resulted in 1.48-fold increase versus the control, C_max_ (357.1 ± 64.7 ng/ml) resulted in 1.38-fold increase versus the control. In the presence of baicalein, the pharmacokinetic parameters of 3,4-CLZ and 4′-CLZ were changed: AUC_0-∞_, C_max_ decreased by 36.1 and 19.5% for 3,4-CLZ, 13.1 and 44.4% for 4′-CLZ, respectively. In addition, CLz/F represented 2.24-fold increase for 3,4-CLZ and 1.77-fold increase for 4′-CLZ compared with those in the controls. These results described above indicated that an apparent herb-drug interaction existed after CLZ combination with baicalein.

## Discussion

Baicalein is not only derived from traditional medicinal plants like root of *Scutellaria baicalensis* (Bai et al., 2020)*,* but also is from foods in daily life (Jin et al., 2017). So it is easy for us to automatically and consciously uptake a certain amount of baicalein. Especially, patients administrated drugs for intervention treatment also eat baicalein-contained medicinal plants or foods in daily life. Therefore, herb-drug interactions between baicalein and other clinical drugs deserve to investigate.

Previous studies showed that baicalein could alter the pharmacokinetic parameters of many clinical proved drugs, when it was combination with other drugs. Hwang, Y. H. et al. found the co-administration of baicalein had an inhibitory effect on the pharmacokinetics of ciprofloxacin and significantly changed the AUC_(0–480 min)_ of single ciprofloxacin (Hwang et al., 2017). Data from two studies also observed that baicalein altered the pharmacokinetics of tamoxifen, and nimodipine in rats (Cho et al., 2011; Li et al., 2011). This research investigated the impact of baicalein on the pharmacokinetics of CLZ and its two metabolites, 3,4-CLZ and 4′-CLZ after oral administration of CLZ (10 mg/kg) with or without baicalein (80 mg/kg) in rats.

Commonly, physiological disposition of drugs *in vivo* contains four processes, including absorption, metabolism, distribution and excretion. Firstly, drugs are absorbed into the blood through the transports like P-glycoprotein, multidrug resistance proteins. CYP450s such as CYP3A4, CYP2C9, CYP2C19 mainly affected and controlled the metabolism of drugs. Transporters such as P-glycoprotein and the multidrug resistance proteins ABCC2 and ABCC3 significantly altered the pharmacokinetics of etoposide in mice (Lagas et al., 2010). Tang pf et al. found that CYP2C9 and CYP3A4 allelic polymorphism could obviously change the ability of sildenafil transforming into N-desmethyl sildenafil (Tang et al., 2020). Therefore, transports and CYP450s plays key roles in the pharmacokinetics of drugs.

In this study, significant changes of pharmacokinetic parameters such as AUC and CLz/F among CLZ, 3,4-CLZ and 4′-CLZ were observed. These results indicated and provided an evidence for the potential herb-drug interaction between baicalein and CLZ in rats. AUC of CLZ markedly increased after combination indicated that baicalein could enhance the oral bioavailability of CLZ. What’s more, AUC_0→∞_ of 3,4-CLZ was almost 5-fold higher than this in 4′-CLZ before combination, which indicated CLZ was more metabolized into the 3,4-CLZ than 4′-CLZ. AUC of 3,4-CLZ and 4′-CLZ significantly decreased after combination implied that baicalein could inhibited the hepatic metabolism of CLZ. We also found that the AUC_0→∞_ ratio of 3,4-CLZ toward 4′-CLZ changed from 5.17 to 3.8, it seemed that metabolic pathway of CLZ metabolized into 3,4-CLZ was more easily to be affected by baicalein than 4′-CLZ. The changes in pharmacokinetic parameters of CLZ, 3,4-CLZ and 4′-CLZ mentioned above might due to the hepatic enzyme and P-glycoprotein. The former study showed that baicalein might suppress the function of CYP3A4 and P-glycoprotein to obviously alter the pharmacokinetics of tamoxifen and nimodipine in rats (Li et al., 2011). Moreover, a previous study showed that the interaction between cilostazol and ambroxol probably due to CYP3A4 (Zhou et al., 2021). A CYP3A4 inhibitor of erythromycin combination with cilostazol could significantly pharmacokinetic parameters of CLZ such as C_max_ and AUC (Suri et al., 1999). These results suggested that CLZ was easy to be affected by CYP3A4 inhibitors. What’s more, Meng, M. et al. found baicalein restrained the enzyme activity of CYP 3A4 in rats liver microsome (Meng et al., 2021). We speculated that baicalein altered pharmacokinetics of CLZ and its metabolites through suppressing CYP 3A4 and P-glycoprotein.

## Conclusion

In conclusion, we established a reliable UPLC-MS/MS method to simultaneously quantify the plasma concentrations of CLZ and its two main metabolites in rats. Meanwhile, a significant influence of baicalein on the pharmacokinetics of CLZ and its metabolites in rats was found in this study. What’s more, there should be a warning notice of the interaction between baicalein and CLZ. Further research should be illustrated the impact of baicalein on the CLZ in human. Preparations containing baicalein should be changed or paid more attentions due to the possibility of herb-drug interactions.

## Data Availability

The original contributions presented in the study are included in the article/Supplementary Material, further inquiries can be directed to the corresponding authors.
